# Supplementary material for: Complexome profiling of the Chlamydomonas psb28 mutant reveals TEF5 as an early PSII assembly factor
Source: Plant Cell. 2025 Mar 17;37(6):koaf055. doi: 10.1093/plcell/koaf055 (PMC12164590; doi:10.1093/plcell/koaf055)
Supplement: koaf055_Supplementary_Data [file koaf055_supplementary_data.zip › psb28_interactive.zip!/plots/intensity/qualityPlots/peptides/psaB_Cre-1.g2717038.t1.p1.html]

### Cre-1.g2717038.t1.p1

Trivial name: psaB  
SeqCoverage [median]: 31.20  
Mass: 82.11 kDa  
  
 

| Sequence | MissedCleavage | Mass [Da] | wtIntensity | muIntensity | Score |
| --- | --- | --- | --- | --- | --- |
| ALYGFDFLLSSK | 0 | 1359.71 | 470822.67 | 228486.75 | 192.12 |
| ATKLFPK | 1 | 803.49 | 4945.55 | 28397.80 | 143.00 |
| ATKLFPKFSQGLAQDPTTR | 2 | 2105.13 | 1158.23 | 0.00 | 31.88 |
| DKPVALSIVQAR | 1 | 1295.76 | 871986.71 | 1151916.26 | 162.38 |
| DYDPEQNK | 0 | 1007.42 | 251563.29 | 140014.14 | 183.90 |
| DYDPEQNKGNVLAR | 1 | 1617.77 | 788532.77 | 714301.58 | 185.95 |
| FSQGLAQDPTTR | 0 | 1319.65 | 1775810.97 | 1287133.64 | 308.21 |
| FSQGLAQDPTTRR | 1 | 1475.75 | 980773.20 | 825746.17 | 194.98 |
| GGASGPVNISTSGVYQWWYTIGMR | 0 | 2586.23 | 24846.19 | 0.00 | 103.24 |
| GYWQELIETLVWAHEK | 0 | 2001.00 | 436.00 | 0.00 | 78.28 |
| IWYGLAMAHDFESHDGMTEENLYQK | 0 | 2984.31 | 114710.21 | 30142.84 | 115.66 |
| LFPKFSQGLAQDPTTR | 1 | 1804.95 | 0.00 | 2085.94 | 51.26 |
| LNHHLSGLFGVSSLAWTGHLVHVAIPESR | 0 | 3133.66 | 86939.58 | 11241.95 | 176.21 |
| MQAILEAHTPPSGSLGAGHK | 0 | 2001.01 | 1136541.85 | 1025823.15 | 206.11 |
| PIAHAIWDPHFGQPAVEAFTR | 0 | 2359.19 | 11182.24 | 6510.91 | 150.61 |
| PVALSIVQAR | 0 | 1052.63 | 365.46 | 0.00 | 77.73 |
| QILIEPVFAQWIQAAHGK | 0 | 2048.12 | 190621.25 | 53330.96 | 258.14 |
| RIWYGLAMAHDFESHDGMTEENLYQK | 1 | 3140.41 | 66518.76 | 17025.61 | 118.59 |
| TNFGIGHR | 0 | 900.46 | 417723.26 | 335774.59 | 196.77 |
| TPLANLVYWK | 0 | 1203.67 | 208568.50 | 66900.68 | 255.54 |
|  |  |  | 7404046.7 | 5924833.0 |  |
